# Mechanistic Study of Failure in CFRP Hybrid Bonded–Bolted Interference Connection Structures under Tensile Loading

**DOI:** 10.3390/ma17092117

**Published:** 2024-04-30

**Authors:** Bin Luo, Liyang Xue, Qingsong Wang, Peng Zou

**Affiliations:** 1 School of Mechanical Engineering, Northwestern Polytechnical University, Xi’an 710072, China; xuely@mail.nwpu.edu.cn; 2 AVIC Chengdu Aircraft Industrial (Group) Company, Chengdu 610041, China; 2020261623@mail.nwpu.edu.cn; 3 Aircraft Strength Research Institute of China, Xi’an 710065, China

**Keywords:** composite material, tensile load, hybrid bonded–bolted, interference fit, failure mechanism

## Abstract

Hybrid bonded–bolted composite material interference connections significantly enhance the collaborative load-bearing capabilities of the adhesive layer and bolts, thus improving structural load-carrying capacity and fatigue life. So, these connections offer significant developmental potential and application prospects in aircraft structural assembly. However, interference causes damage to the adhesive layer and composite laminate around the holes, leading to issues with interface damage. In this study, we employed experimental and finite element methods. Initially, different interference-fit sizes were selected for bolt insertion to analyze the damage mechanism of the adhesive layer during interference-fit bolt installation. Subsequently, a finite element tensile model considering damage to the adhesive layer and composite laminate around the holes post-insertion was established. This study aimed to investigate damage in composite bonded–bolted hybrid joints, explore load-carrying rules and failure modes, and reveal the mechanisms of interference effects on structural damage and failure. The research results indicate that the finite element prediction model considering initial damage around the holes is more effective. As the interference-fit size increases, damage to the adhesive layer transitions from surface debonding to local cracking, while damage to the composite matrix shifts from slight compression failure to severe delamination and fiber-bending fracturing. The structural strength shows a trend of initially increasing and then decreasing, with the maximum strength observed at an interference-fit size of 1.1%.

## 1. Introduction

Carbon-fiber-reinforced polymer/plastic composite materials, known for their low specific modulus, high strength, and excellent fatigue performance, have become one of the most widely used aerospace materials. In large composite structures, connecting composite materials with other relevant materials is a necessity. The most commonly used methods for connecting composite materials in industrial settings are adhesive bonding and using bolted connections and hybrid connections [1,2]. Compared to traditional bolted and adhesive connections, hybrid bonded–bolted connections can enhance structural connection performance such that is meets the requirements of aerospace equipment [3,4,5]. However, in hybrid bonded–bolted connections, there is a significant disparity in stiffness between the adhesive layer and the bolts, resulting in minimal shared load-carrying capacity between them when structural loading is applied [6,7,8]. This substantially reduces the efficiency of the connection structure, attracting widespread attention from scholars in the aerospace field [9,10,11]. Research has revealed that bolt-hole interference fit plays a pivotal role in addressing the issue of low bolt load-carrying efficiency in hybrid bonded–bolted connections. Interference connections not only improve the fatigue performance of the structure but also increase the contact area around the pinhole [12,13,14], effectively enhancing the load-carrying efficiency of the bolts in hybrid bonded–bolted connection structures, thereby enhancing the ultimate strength of the structure [15,16]. Due to fastener and manufacturing process limitations, during the interference fit process, the adhesive layer and the composite material around the hole are subjected to axial forces from the bolts. This generates radial compressive forces and axial friction forces at the pinhole interface, resulting in a complex stress field between the composite material, adhesive layer, and bolts. As the axial forces nonlinearly increase, the stress field changes rapidly, leading to potential damage to the adhesive layer and the composite material around the hole. This damage at the connection interface is exacerbated under tensile loading, as hole perimeter damage evolves and propagates, causing premature structural failure. This significantly impacts the load-carrying performance of the connection structure and severely limits the development of hybrid bonded–bolted connection technology.

Research in the aerospace industry on hybrid bonded–bolted composite connections can be traced back to the work of L.J. Hart-Smith in the 1980s [17,18]. Initially, the primary objective was to use these connections for structural reinforcement and repair to enhance damage tolerance. To overcome the limitations of adhesive joints, mechanical fastening was introduced, resulting in hybrid joints. Previous studies have demonstrated that the mechanical fasteners in hybrid connections can prevent catastrophic failures associated with adhesive bond failures, ensuring structural safety. Numerous domestic and international researchers have conducted extensive studies on the failure and damage analysis of hybrid bonded–bolted connection structures [19]. Research has shown that stress levels around the hole perimeter and the extent of damage increase with an increase in interference [20,21]. In recent years, with the development of composite material mechanics, progressive damage analysis methods have become essential for predicting the load-carrying capacity and failure behavior of composite connection structures [22]. The three-dimensional Hashin failure criteria have been widely applied in damage prediction studies of composite structures [23,24]. Researchers have analyzed the load response behavior of composite bonded–bolted connection structures by establishing failure criteria that characterize different damage modes and mechanical models simulating the initiation and propagation of adhesive layer cracks, thereby revealing their damage and failure mechanisms [25]. Raphael Blier et al. [26] conducted a study on the tensile performance of hybrid bonded–bolted joints using finite element analysis. The tensile performance of the hybrid joints was found to be significantly superior to that of bolted joints. Paliwal et al. [27] investigated the failure mechanisms of CFRP bolted, bonded, and hybrid joints under quasi-static loading conditions, suggesting that hybrid joints exhibit stronger damage tolerance. Li et al. [28] demonstrated that hybrid joints exhibit higher peak loads and energy absorption than bolted joints under both static and dynamic loading conditions. Furthermore, for hybrid joints, the addition of the adhesive layer changed the failure mode of the GFRP material from shear-out failure to tension failure. Zuo et al. [29] employed an electromagnetic dynamic connection method to investigate the damage behavior of structures during the interference bolt installation process. Hu et al. [30] established three-dimensional failure criteria and exponential damage evolution criteria accounting for the influence of temperature effects, studying the damage interface and preload response under different levels of interference. Yan et al. [31] conducted a study on the damage caused by bolts during the insertion process of hybrid bonded–bolted interference connections, but the influence of the damage generated after insertion on the structure’s performance was not considered. Progressive damage in the adhesive layer generally corresponds to Mode I, Mode II, and mixed-mode (Mode III) fractures. Cohesive Zone Models (CZMs) based on damage mechanics are mainly used to analyze the initiation and propagation of adhesive layer damage and cracks [32,33].

Furthermore, for the hybrid bonded–bolted (HBB) joints in composite materials, the damage to the adhesive layer and around the composite material holes caused during installation should also be considered in subsequent tensile loading models. The influence of initial damage to the adhesive layer and around the holes on structural strength cannot be ignored. Existing studies have focused on separate processes of the installation and tensile loading of bonded–bolted joints, with limited investigation into the structural damage caused by bolt installation and its effects on structural strength and failure modes. In this paper, an improved three-dimensional Hashin damage initiation criterion, the Camahno material property degradation criterion, and a cohesive zone model with a bilinear constitutive behavior were employed to establish a finite element model for hybrid bonded–bolted joints considering initial damage around the holes and adhesive layer. Combined with tensile failure tests, this study analyzed the tensile deformation of structural components and the microscopic morphology of bolt-hole interference interfaces, investigated the bearing strength of hybrid connections with different interference fits, and revealed the mechanisms of structural damage and failure. By assessing the tensile failure behavior of hybrid bonded–bolted joints in composite materials, this research provides guidance for the design and application of such joint structures.

## 2. Numerical Simulation

### 2.1. Construction of a Finite Element Model

**Figure 1:**
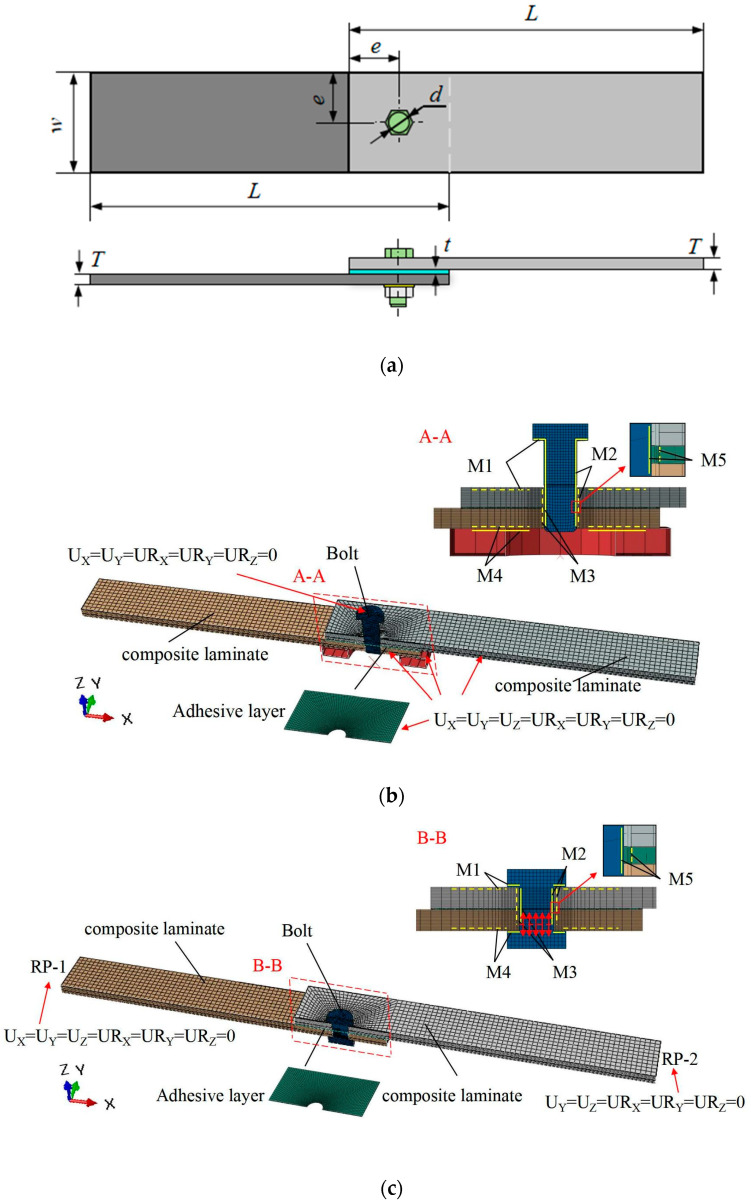
Establishment of a finite element model for composite material adhesive–bolt hybrid interference connection structure: (**a**) geometric model, (**b**) installation process, and (**c**) tensile process.

First, we establish a geometric model of the hybrid bonded–bolted interference-fit structure. As shown in Figure 1a, the geometric model of the composite laminate has a length L of 135 mm and a base diameter d of 6 mm. The width-to-diameter ratio (w/d) of the carbon fiber composite laminate was set to 6, and the end-to-diameter ratio (e/d) was set to 3; thus, W = 36mm, and e = 18 mm. The adhesive layer thickness t was designed to be 0.2 mm. The construction of the finite element model for the composite material bonded–bolted hybrid interference connection structure involves two distinct phases: the installation process and the tensile process. As illustrated in Figure 1b, the finite element model for the installation of the composite material adhesive–bolt hybrid interference connection structure comprises upper and lower composite laminate plates, an adhesive layer, bolts, and support plates. The carbon fiber composite laminate plates and bolts were discretized using eight-node reduced integration element C3D8R for meshing, while the adhesive layer was represented by cohesive element COH3D8. As depicted in Figure 1c, the finite element model for the tensile process of the composite material adhesive–bolt hybrid interference connection structure encompasses upper and lower composite laminate plates, an adhesive layer, and bolts. Similarly, the composite laminate plates were meshed using C3D8R element types, bolts were discretized using eight-node reduced integration element C3D8R, and the adhesive layer was characterized using cohesive element COH3D8.

During the installation process, the boundary conditions for the upper and lower composite laminates, adhesive layer, and support plate were set as fixed constraints. The bolt was assigned a target displacement in the UZ direction to simulate the installation process of the composite bonded–bolted interference-fit joint structure. In the tensile process, in the finite element model, the boundary conditions for the fixed end of the lower composite laminate were set as fixed constraints, while the boundary conditions for the loading end of the upper composite laminate were set as UY = UZ = URX = URY = URZ = 0. Contact relationships were defined as follows: apart from the bonding between the adhesive layer and the carbon fiber composite laminate, the normal behavior between the bolt, adhesive layer, carbon fiber composite laminate, and support plate was modeled using hard contact properties to prevent mesh penetration. Tangential behavior was modeled using a penalty-based contact friction model. As shown in Figure 1, solid lines represent master surfaces, while dashed lines represent slave surfaces. A total of five contact pairs were established, including M1 (bolt head–upper laminate), M2 (bolt shaft–upper-laminate-hole wall), M3 (bolt shaft–lower-laminate-hole wall), M4 (support plate–lower laminate), and M5 (bolt shaft–adhesive layer). The friction coefficient was set to 0.3 for contact pairs M1 and M4 and 0.1 for all other contact surfaces. The friction coefficient between the upper and lower composite laminates was set to 0.4.

The composite laminate used in the tensile failure test of the hybrid bonded–bolted joint structure was provided by Weihai Guangwei Composites Co., Ltd., Weihai, China. The laminate was prepared using a vacuum bag molding process, and its prepreg consisted of T700/3660 carbon fiber–epoxy resin prepreg. The nominal thickness of a single composite laminate was 3.6 mm, with a stacking sequence of [0°/45°/−45°/90°] 3s and a nominal ply thickness of 0.15 mm. These laminates’ mechanical properties are summarized in Table 1. Following the ASTM D5961 testing standard [34], all composite laminate sheets were cut into specimens with geometric dimensions of 135 mm × 36 mm using water-cutting equipment. The fastener used during the interference-fit connection process was a titanium alloy bolt. To ensure the accuracy and efficiency of the finite element analysis, the threaded portion of the bolt was simplified. The bolt shank diameter was 6 mm, and the material was (TC4) Ti-6Al-4V; their mechanical properties are provided in Table 2. For the adhesive process, Ergo7200 epoxy structural adhesive (Kingbond Adhesive Co., Ltd., Shenzhen, China.) was used as the adhesive layer material, and its mechanical properties are detailed in Table 3.

### 2.2. Failure Criteria and Material Property Degradation Rule

During the loading process, carbon fiber composite materials are subjected to both in-plane and out-of-plane stresses, resulting in various failure modes, including fiber damage, matrix damage, and interlaminar delamination. In this study, an analysis of damage in the elements was carried out in accordance with the maximum stress criterion. An adapted three-dimensional Hashin criterion was employed to predict different damage modes in composite materials. Additionally, the Ye delamination criterion was utilized to assess interlaminar tensile and compressive failures under the influence of interlaminar tensile and compressive stresses [37], which are explained in detail as follows:

Fiber tensile failure ($\sigma_{11}\geq 0$):

(1) $$F_{ft}={\left(\frac{\sigma_{11}}{X_{t}}\right)}^{2}$$

Fiber compression failure ($\sigma_{11}<0$):

(2) $$F_{fc}={\left(\frac{\sigma_{11}}{X_{c}}\right)}^{2}$$

Matrix tensile failure ($\sigma_{22}$ + $\sigma_{33}\geq$ 0):

(3) $$F_{mt}={\left(\frac{\sigma_{12}}{S_{12}}\right)}^{2}+{\left(\frac{\sigma_{13}}{S_{13}}\right)}^{2}+{\left(\frac{\sigma_{22}+\sigma_{33}}{Y_{t}}\right)}^{2}+\frac{\sigma_{23}^{2}-\sigma_{22}\sigma_{33}}{S_{23}^{2}}$$

Matrix compressive failure ($\sigma_{22}$ + $\sigma_{33}<$ 0):

(4) $$F_{mc}={\left(\frac{\sigma_{12}}{S_{12}}\right)}^{2}+{\left(\frac{\sigma_{13}}{S_{13}}\right)}^{2}+{\left(\frac{\sigma_{22}+\sigma_{33}}{2S_{23}}\right)}^{2}+\frac{\sigma_{23}^{2}-\sigma_{22}\sigma_{33}}{S_{23}^{2}}+\frac{\sigma_{22}+\sigma_{33}}{Y_{c}}\left[{\left(\frac{Y_{c}}{2S_{23}}\right)}^{2}-1\right]$$

Fiber-matrix shear failure ($\sigma_{11}<0$):

(5) $$F_{ms}={\left(\frac{\sigma_{11}}{X_{C}}\right)}^{2}+{\left(\frac{\sigma_{12}}{S_{12}}\right)}^{2}+{\left(\frac{\sigma_{13}}{S_{13}}\right)}^{2}$$

Delamination in tension ($\sigma_{33}<0$):

(6) $$F_{nt}={\left(\frac{\sigma_{33}}{Z_{t}}\right)}^{2}+{\left(\frac{\sigma_{13}}{S_{13}}\right)}^{2}+{\left(\frac{\sigma_{23}}{S_{23}}\right)}^{2}$$

Delamination in compression ($\sigma_{33}<0$):

(7) $$F_{nc}={\left(\frac{\sigma_{33}}{Z_{c}}\right)}^{2}+{\left(\frac{\sigma_{13}}{S_{13}}\right)}^{2}+{\left(\frac{\sigma_{23}}{S_{23}}\right)}^{2}$$

$\sigma_{11}$ represents stress in the fiber direction, while $\sigma_{22},\sigma_{33}$ represent stress in the transverse direction and in the thickness direction. $\tau_{12}$, $\tau_{23},\;\tau_{13}$ represent shear stress. $X_{t}$ represents tensile strength in the fiber direction, $X_{c}$ represents compressive strength in the fiber direction, $Y_{t}$ represents tensile strength in the transverse direction, $X_{c}$ represents compressive strength in the transverse direction, $Z_{t}$ represents tensile strength in the thickness direction, $Z_{c}$ represents compressive strength in the thickness direction, and $S_{12},\;S_{13},\;S_{23}$ represent shear strength. Damage occurs in the carbon fiber composite material element when $F_{ft}$, $F_{fc}$, $F_{mt}$, $F_{mc}$, $F_{ms}$, $F_{nt}$, and $F_{nc}\geq 1$.

As shown through stress analysis, once the stress state of the composite material element satisfies the corresponding damage criteria mentioned above, the elastic modulus, shear modulus, and the Poisson’s ratio of the element experience a partial decline. The stiffness of the composite material element is considered to be reduced to a certain value through a stiffness reduction coefficient, known as the partial degradation model. Once this was accounted for, the simulation calculation then continued. Referring to the degradation criteria proposed by Camanho [38], Matthews, Tserpes, and others, necessary modifications were made after performing trial calculations and observing damage distribution. The degradation behavior can be expressed as follows, and the degree of degradation is shown in Table 4.

### 2.3. Finite Element Simulation Method and Workflow

The prediction of damage behavior during the installation of composite material adhesive–bolt hybrid interference connection structures includes damage to the adhesive layer and in-plane as well as out-of-plane damage in the composite material. For the prediction of damage in the adhesive layer, an internal cohesive model embedded in the ABAQUS 2020 finite element software product was employed, and the constitutive parameters for the adhesive layer material were custom-defined. Damage states were characterized using a stiffness degradation index, which was stored in SDEG. The stiffness degradation index indicates the damage state, ranging from 0 (undamaged) to 1 (failed). When adhesive layer elements fail, the elements are removed. Regarding in-plane and out-of-plane damage in the composite material, a material subroutine (UMAT) was developed and written in FORTRAN to perform stress analysis on carbon fiber composite materials. Stress analysis was conducted on adhesive layer and composite material elements based on failure criteria for different damage modes. This included stiffness matrix, damage criteria, and property degradation criteria, all of which were generated using ABAQUS.

**Figure 2:**
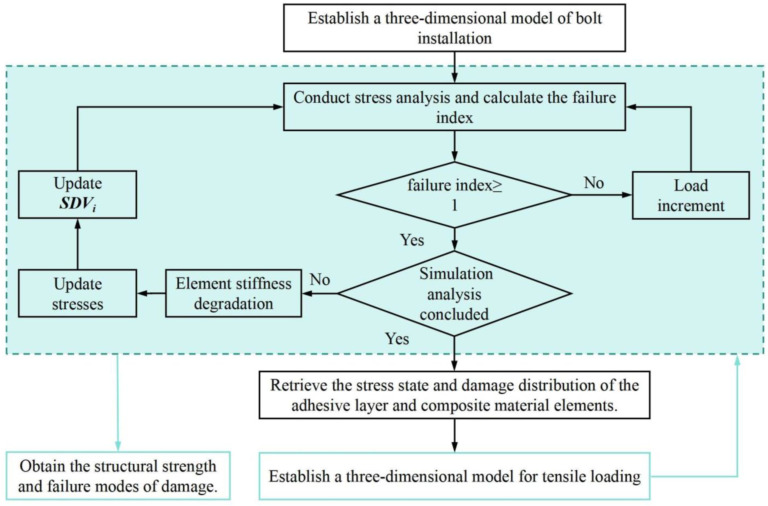
Finite element model analysis workflow.

A failure index less than 1 indicates that a material is in the linear elastic deformation stage and the load has not reached the material’s damage initiation load. When the failure index reaches 1, if the simulation analysis process is not terminated, the corresponding damage variables are activated. The material elements are used to calculate and update the stress state, stiffness matrix, and state variables (SDVi) of the material elements based on the property degradation criteria. Then, an incremental step is executed until the bolt installation process satisfies the boundary conditions consistent with the test conditions or the material reaches a failure state. At this point, the simulation analysis process concludes, and the distribution of damage in the adhesive layer and around the composite material holes is determined. This information is then used to establish a three-dimensional model for tensile loading, followed by another round of simulation analysis to determine the structural strength and damage failure modes. The workflow for the finite element model application is illustrated in Figure 2.

**Figure 3:**
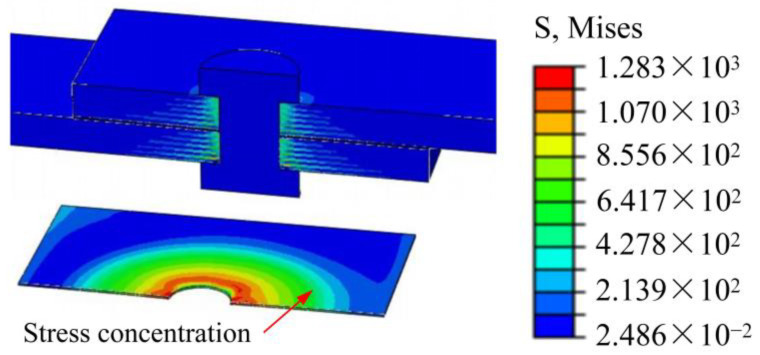
The residual stresses around the connection holes after bolt installation.

In practical situations, due to the compressive and frictional effects of bolts, it is inevitable that damage will occur around the adhesive layer and composite material holes. Therefore, in the finite element model calculations of composite material adhesive–bolt hybrid interference connection structures under tensile loading, it is necessary to consider the initial damage caused by interference fit during installation. In the tensile finite element model, the installation and tensile loading processes of the connection structure can be analyzed by establishing multiple analysis steps. However, each subsequent analysis step and the establishment of boundary conditions are influenced by the previous analysis step. After bolt installation is completed, it is difficult to address the issue of applying bolt pre-tightening forces directly. To overcome this challenge, the ‘Initial state’ command in the predefined field is used to transfer the stress, deformation, and damage results from the finite element model of bolt installation (as shown in Figure 3) to the tensile finite element model in its initial state. This allows for the continuation of subsequent analyses. Figure 3 illustrates the distribution of residual stresses at the interference connection site after the bolts have been installed and before simulating the tensile behavior of the composite material adhesive–bolt hybrid interference connection structure.

## 3. Tensile Failure Testing and Finite Element Model Validation

### 3.1. Experimental Test Plan

In accordance with the ASTM D5961 testing standard [34], all composite laminate specimens were cut using water-cutting equipment to geometric dimensions of 135 mm × 36 mm. Different interference-fit sizes were created by using differently sized tools to create holes of varying diameters. The nominal diameter (D) of the titanium alloy bolt shank was 6 mm, but the actual diameter was adjusted based on measurements to match the different hole sizes in the composite laminate, ensuring the desired interference-fit size. In this study, we plan to establish four interference levels, defining interference level as follows [39]:

(8) $$I_{r}=\frac{D-d}{d}\times 100\%$$

Here, D and d are the nominal diameters of the bolt shank and the connection hole in the composite material laminate, respectively. According to recommended interference fit dimensions in engineering applications, we selected interference levels of 0.0%, 0.5%, 1.1%, and 2.0%. Consequently, the corresponding connection hole diameters were d_1_ = 5.97 mm, d_2_ = 5.93 mm, and d_3_ = 5.88 mm. Four test groups were established, with concurrent bolted joint control tests conducted under the same interference-fit conditions.

To achieve the desired interference-fit size, custom drill bits with corresponding diameters were used to create integrated hole-cutting tools. The composite laminate was drilled using a coordinate measuring machine (JDHGT 400 A10H, Beijing Jingdiao Technology Co., Ltd., Beijing, China) at a tool speed of 2200 r/min and a feed rate of 2 mm/min. After drilling, the actual diameters of the holes and bolts were measured using a high-precision caliper (with an accuracy of 0.01 mm). This allowed for one-to-one matching based on the hole and bolt diameters, and any non-compliant specimens were identified and discarded due to machining deviations and part tolerances. The specimens were then fixed on custom fixtures for adhesive application and curing at room temperature for 72 h. The adhesive layer thickness was selected within the recommended range at 0.2 mm [7]. After the specimens were cured, the interference-fit process was initiated. A universal testing machine (INSTRON, 10 kN, Instron Corporation, Boston, MA, USA) was used to vertically insert titanium alloy bolts into the composite adhesive specimens at a rate of 1 mm/min. Following bolt insertion, professional-grade torque wrenches were used to tighten the washers and nuts to a predetermined torque setting of 3.6 Nm, completing the interference-fit process for the composite test specimens.

To ensure the reliability of the test results, according to the above specimen fabrication plan, three specimens were prepared for each of the four different interference fits for testing. Each test was repeated three times, and the load–displacement curves were recorded for each test run. After excluding obvious erroneous test results, the collected data points from each test were averaged to obtain the required load–displacement curves. Finally, the interface micro-damage behavior of the failed specimens was observed using a high-depth-of-field 3D microscope. The average of the three test results was calculated to determine the tensile failure load for the hybrid bonded–bolted joints under different interference fits compared to bolted joints. Line graphs were then plotted to visually compare the effects of interference fit on structural strength.

### 3.2. Experimental Procedure

**Figure 4:**
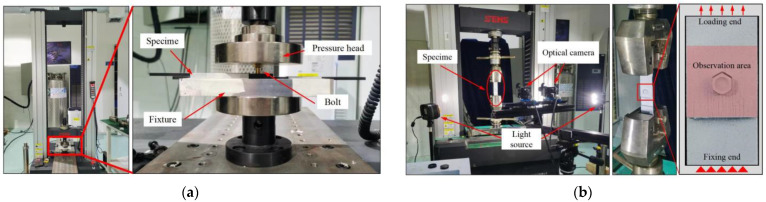
Structural component testing platform; (**a**) installation process; (**b**) tensile process.

The initial step involved performing pin insertion for both the adhesive–bolt hybrid and bolt interference connections. This procedure was carried out using an INSTRON 10 kN universal testing machine, as depicted in Figure 4a. To prevent any sliding of the upper composite laminate during the bolt insertion process, an appropriately sized spacer was positioned beneath it. At the bottom of the lower laminate, a fixed fixture with a central hole with a radius of 5 mm was placed to prevent any interference or collision with the base during bolt insertion. Since the dimensions of the bolt shank are larger than the diameter of the connection hole, the threaded segment of the bolt could be manually placed within the upper plate to ensure minimal tilting of the bolt in relation to the connection hole. Subsequently, the pressing head was lowered to the surface of the bolt head. The feed rate of the pressing head was set at 1 mm/min, allowing for a gradual insertion of the bolt into the connection hole, effectively completing the pin insertion process. When the lower surface of the bolt head made contact with the upper surface of the composite laminate, there was a noticeable abrupt increase in the pinning load, signaling the termination of the test. This entire testing procedure was replicated three times for each test group, and detailed records of the pinning load–displacement curves throughout the tests were meticulously documented. As a final step, the specimens were sectioned and meticulously polished in the fiber direction up to the center of the bolt. Subsequently, they were subjected to examination for micro-damage behavior at the interface using an advanced deep-focus three-dimensional microscope (Hirox RX-100, Shanghai Hirox Instrument Technology Co., Ltd., Shanghai, China).

The tensile-testing process was conducted using a high-capacity universal testing machine (INSTRON, 100 kN). Simultaneously, a 3D Digital Image Correlation (DIC) system (ARAMIS, GOM, GOM GmbH, Braunschweig, Germany) was employed to measure the surface strain distribution and out-of-plane deformations of the structural components, as illustrated in Figure 4b. To ensure synchronized data acquisition, the load data from the testing machine were incorporated into the 3D DIC system via a synchronized data transfer device. To ensure accurate data collection, the image capture frequency of the 3D DIC system was set to 1 Hz. All specimens were loaded at a crosshead rate of 2 mm/min until complete failure of the specimens occurred. The load–displacement curves during the testing process were recorded, and each test was repeated three times. Finally, the failed specimens were once again subjected to examination using an advanced deep-focus three-dimensional microscope to observe the micro-damage behavior at the interface.

### 3.3. Finite Element Model Validation

**Figure 5:**
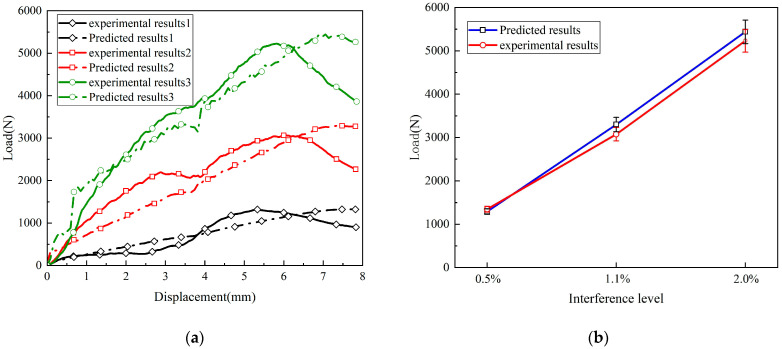
Finite element analysis and experimental load–displacement response for the hybrid bonded–bolted interference bolt installation process: (**a**) Comparison of load–displacement curves; (**b**) comparison of ultimate loads.

In Figure 5, a comparison between the established finite element models and the experimentally obtained load–displacement curves is presented. It can be observed that the overall trends of the finite element model’s calculations align well with the experimental results. The maximum applied loads for interference fits of 0.5%, 1.1%, and 2.0% were compared to the experimental results, resulting in relative errors of 4.07%, 5.34%, and 3.96%, respectively. Therefore, the overall mechanical response of the composite material adhesive–bolt hybrid interference connection structure during the installation process can be predicted accurately.

**Figure 6:**
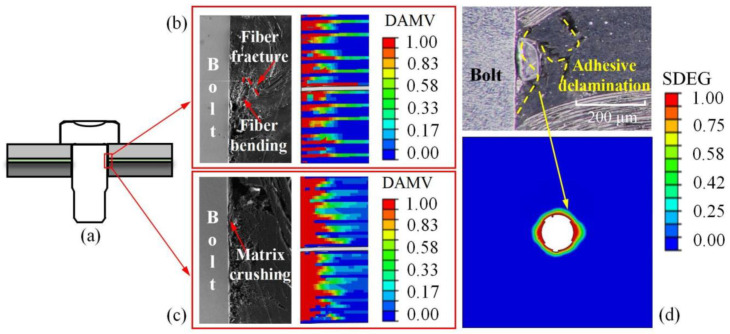
Comparison between the finite element model results and experimental results of the contact interface after bolt installation: (**a**) observed area, (**b**) fiber compression damage, (**c**) matrix compression damage, and (**d**) adhesive layer damage.

As shown in Figure 6, a comparison was made between the interference connection interface damage behavior predicted by the finite element model after bolt installation and the results of the bolt installation test. The observed area of the interference connection interface is shown in Figure 6a. During the downward movement of the bolt, the fiber and matrix mainly suffered damage due to compressive stress, with less damage caused by tensile stress. Figure 6b,c show fiber fracture and matrix crushing, respectively, at an interference level of 2.0%. Damage variables of 0 indicate undamaged material, while 1 indicates material failure. It was observed that the extent of matrix damage was significantly greater than fiber damage. When the bolt passed through the adhesive layer area, the adhesive layer in the interference area was damaged. This was due to axial stress exceeding the peel strength of the adhesive layer. The finite element model accurately predicted this failure mode, as shown in Figure 6d, where the adhesive layer had experienced stiffness degradation, and some elements were deleted. Therefore, the finite element model established in this study can accurately predict the contact interface damage behavior of the adhesive–bolt hybrid interference connection structure.

**Figure 7:**
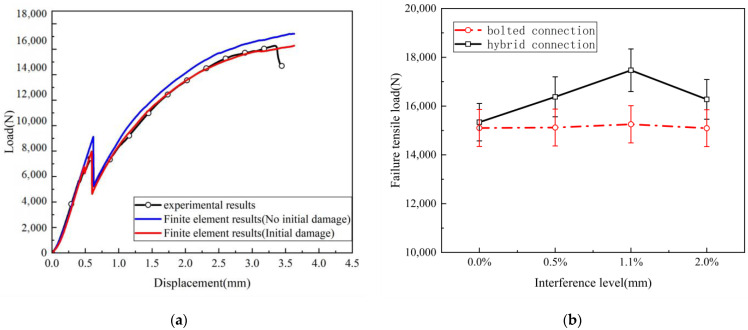
The tensile load–displacement response curves of finite element models and experiments: (**a**) hybrid interference-fit connection structures; (**b**) comparison of failure tensile loads under different connection methods.

Figure 7a illustrates the finite element model and experimental tensile load–displacement response curve when the interference fit is 2.0%. All the curves exhibit a high degree of consistency in their trends, indicating that the results yielded by the finite element tensile model align with the tensile behavior of the test specimens. Additionally, material defects and process errors caused by factors such as curing bubbles, adhesive layer thickness, hole-drilling burn, etc., led to dispersion in the first peak load when the adhesive layer completely failed, resulting in significant differences in ultimate tensile load and failure displacement. Further comparison reveals that initial damage has an impact of up to 13.97% on the adhesive layer failure load and 6.63% on the composite material failure load. This indicates that considering the initial damage to the adhesive layer and the area around the composite material during the installation process results in tensile failure loads that are more akin to the experimental results, and the finite element model should account for the initial damage to the adhesive layer and the composite material. Furthermore, the failure mode and region also exhibit good consistency with the experimental results.

Figure 7b compares the tensile failure loads of hybrid bonded–bolted (HBB) joints and bolted joints at different interference-fit sizes. The experimental results show that, compared to bolted interference-fit structures, the hybrid bonded–bolted (HBB) joints exhibit an increased load-bearing capacity at interference-fit sizes of 0.0%, 0.5%, 1.1%, and 2.0%, with enhancement ratios of 1.57%, 8.32%, 14.5%, and 7.83%, respectively. The maximum failure load occurs at an interference-fit size of 1.1%, followed closely by sizes of 2.0% and 0.5%, which exhibit similar failure loads. The tightest fit results in the smallest failure load. Conversely, the bolted interference-fit joints exhibit nearly identical tensile failure loads across all interference-fit sizes. This indicates that excessive interference-fit sizes can significantly compromise the effectiveness of structural strength enhancement. Therefore, the optimal interference-fit size identified in this study is 1.1%.

**Figure 8:**
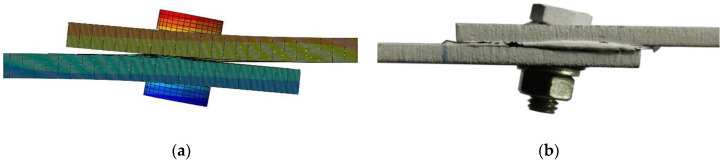
Comparison of out-of-plane deformation in the structural component: (**a**) finite element results; (**b**) experimental results.

The twisting of the bolt resulted in the division of the connection hole wall into the primary load-bearing zone and the secondary load-bearing zone, causing the upper- and lower-layer laminates to bear loads on different planes, leading to additional out-of-plane deformation until failure. The finite element simulation and tensile test results are shown in Figure 8a,b respectively. As shown, this model can effectively predict the failure modes and mechanical responses of composite adhesive–bolt hybrid interference connection structures. Additionally, in the tensile finite element model, a feature was established to delete adhesive layer elements when the stress on the element exceeds its strength, causing the element to immediately fail and be removed.

## 4. Analysis of Failure Mechanisms

### 4.1. Impact of Interference on Stress Distribution in the Adhesive Layer

The interference fit between the adhesive layer and the composite material hole wall due to the action of a bolt results in highly non-uniform residual stresses. The distribution and nature of these stresses are extremely complex, especially under tensile loads. Stress concentration leads to premature failure of the adhesive layer, weakening the synergistic load-bearing effect between the adhesive layer and the bolt, thereby reducing structural load-bearing performance. Therefore, it is necessary to conduct stress analysis of the adhesive layer.

**Figure 9:**
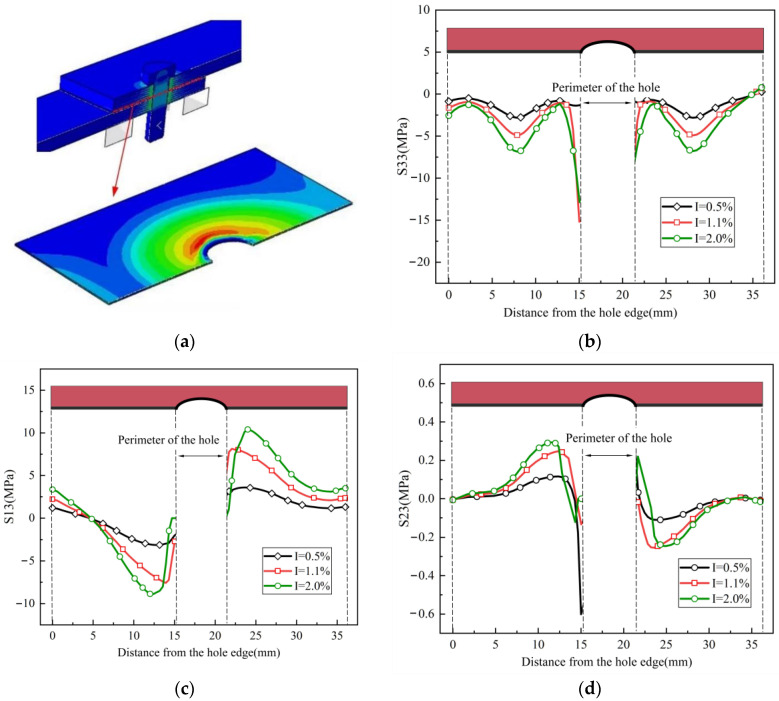
Stress distribution in the adhesive layer after bolt installation: (**a**) stress distribution, (**b**) peel stress S33, (**c**) shear stress S13, and (**d**) shear stress S23.

The stress distribution in the adhesive layer after bolt installation is shown in Figure 9a. Figure 9b depicts the distribution of peel stress S33 along the center line of the adhesive layer after bolt installation, while S13 and S23 represent the two shear stresses in the x-z and y-z directions of the adhesive layer, as shown in Figure 9c,d, respectively. It can be observed that S33 exhibits a sinusoidal variation along the center line, with a sharp increase near the adhesive layer hole. The value of S23 is quite small, while S13 follows a similar pattern to S33 but does not exceed the shear strength of the adhesive layer, indicating that S33 is the primary form of stress causing adhesive layer damage. It is worth noting that when the interference fit was 2.0%, some elements near the adhesive layer hole exhibited a stiffness degradation of 0, and stress could not be captured. Therefore, S33 at 1.1% interference is higher than that at 2.0%.

**Figure 10:**
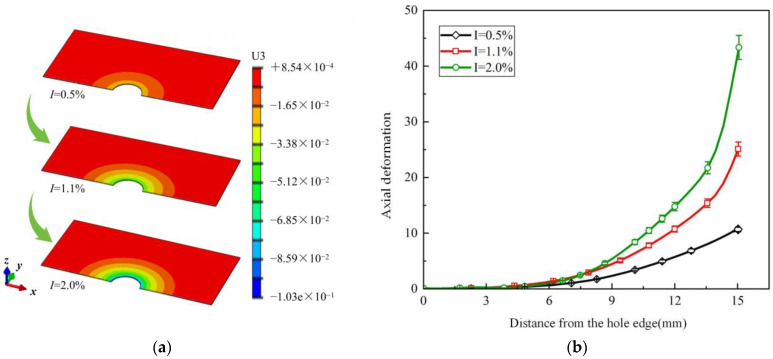
The effect of interference on axial deformation of the adhesive layer: (**a**) deformation contour plot around the hole; (**b**) axial deformation rate.

The impact of interference fit led to axial deformation around the adhesive layer, and the results of the finite element simulation align well with experimental results. The axial deformation was calculated along the center line, and it increases when moving closer to the adhesive layer hole. Furthermore, as the interference fit increases, the contact between the bolt and hole becomes tighter, leading to greater axial deformation in the adhesive layer. Figure 10a,b display the statistical analysis of the deformation cloud distribution and the axial deformation rate around the adhesive layer hole for different interference fits, with maximum deformation rates of 10.8%, 25.2%, and 44.1%, respectively. It can be observed that in the case of I = 2.0%, there is a steep increase in axial deformation around the adhesive layer hole, and excessive deformation can result in damage to the adhesive layer material, thereby inducing crack initiation. Therefore, selecting an appropriate interference fit along with an adhesive layer material with a sufficient level of toughness can reduce damage.

Due to the compressive action of the interference bolt, initial damage around the composite material hole is inevitably generated, especially with excessive interference fit, which can lead to severe adhesive layer damage, matrix crushing, and fiber fracture. This significantly reduces the benefits of the adhesive–bolt hybrid interference connection structure. Although a considerable amount of research has been conducted on the damage generated around composite material holes during interference fit processes, further investigation is still needed to analyze adhesive layer damage during bolt installation processes.

**Figure 11:**
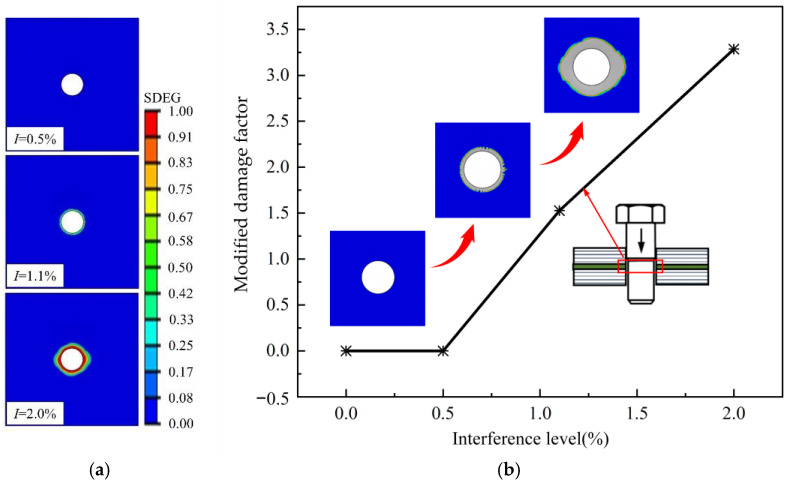
The influence of interference on damage around the adhesive layer: (**a**) hole edge stiffness degradation; (**b**) adhesive layer damage factor.

In order to visually analyze the damage area of the adhesive layer after bolt installation had been completed, mesh deletion was turned off. The final adhesive layer damage situation is shown in Figure 11a. A stiffness degradation index of 0 indicates that the adhesive layer elements have not experienced stiffness degradation, while a value of 1 indicates complete stiffness degradation. When the interference fit is 0.5%, the adhesive layer elements are almost undamaged. When the interference fit is 1.1%, damage starts to occur around the adhesive layer hole, but adhesive layer elements do not fail. When the interference fit is 2.0%, severe cohesive damage occurs near the adhesive layer hole, and the adhesive layer elements are severely damaged.

The damaged elements are defined as the sum of deleted elements (completely damaged) and partially damaged elements, where partially damaged elements refer to adhesive layer elements with a stiffness degradation index greater than 0.1. The degree of damage to adhesive layer elements around the hole is characterized by a damage factor, as shown in Figure 11b. The gray area represents the damaged region of adhesive layer elements. It can be observed that when the interference fits are 0.0% and 0.5%, the damage factor for adhesive layer elements is 0, indicating that no damage has been inflicted on these elements at this point. When the interference fit is 1.1%, there are 288 damaged adhesive layer elements with a modified damage factor of 1.53, and the damaged area is relatively small. However, when the interference fit is 2.0%, the number of damaged elements increases to 786, and the damage to elements around the adhesive layer hole rapidly expands outward, with a modified damage factor reaching 3.29. This indicates that a larger interference fit leads to significant initial installation damage in the adhesive layer. Therefore, choosing an appropriate interference fit range is crucial, and considering the initial damage around the hole during structural component tensile simulation is reasonable.

### 4.2. Impact of the Adhesive Layer on Stress Distribution around the Hole

**Figure 12:**
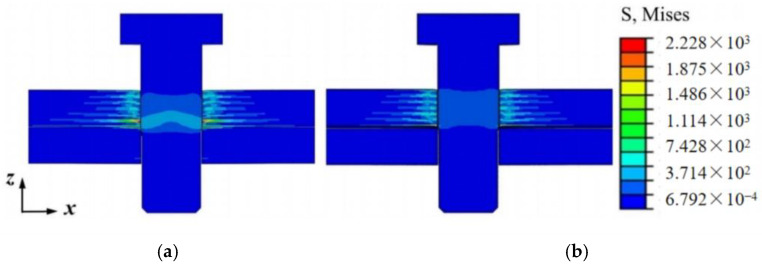
Compressive stress distribution around the composite material hole corresponding to bolts: (**a**) bolted connection; (**b**) hybrid connection.

The adhesive layer provides strong constraints in the overlapping area of the composite upper- and lower-laminate plates, which differs from the case for bolted connections. In the case of bolted connections, the compression force exerted by the bolts undergoes changes. Compression-induced stress concentration around the holes in the composite material occurs and directly affects the damage area and state at the interference contact interface. Under tensile loads, the damage area evolves and expands, accelerating the damage to the connection structure. To further analyze the influence of the adhesive layer on the stress distribution around the holes in the composite laminate, this section compares the stress distribution around the composite material holes when the bolt shank is pressed against the upper laminate and when it is inserted into the upper laminate, as shown in Figure 12, based on finite element analysis results.

**Figure 13:**
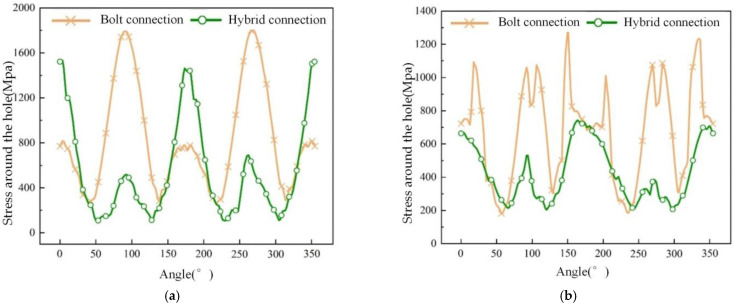
Stress distribution around the hole when the bolt passes through the upper and lower laminate: (**a**) upper laminate; (**b**) lower laminate.

For both interference fit and bolted connections with an interference level of 2.0%, the stress distribution around the holes in the upper- and lower-laminate plies with a 0° orientation, near the adhesive layer, is shown in Figure 13. The stress distribution around the composite laminate holes was obtained through post-processing.

Regarding the upper-laminate holes with a 0° orientation, there is a clear and consistent pattern in the stress distribution between the bolted and adhesive connections, as shown in Figure 13a. It can be observed that the minimum stress value for the adhesive connection (103.8 MPa) is lower than that for the bolted connection (273.7 MPa), while the maximum stress value for the bolted connection (1805.7 MPa) is higher than that for the adhesive connection (1534.7 MPa). Compared to the bolted connection, the stress amplitude is significantly reduced along the hole circumference from approximately 50° to 150° and from about 225° to 325° for the adhesive connection.

For the lower laminate holes with a 0° orientation, there is some fluctuation in the stress distribution around the hole circumference, indicating that excessive interference fit eventually leads to extensive material damage or even failure around the hole, resulting in computational instability, as shown in Figure 13b. Overall, the stress distribution around the hole for the adhesive connection has a significantly lower amplitude compared to that of the bolted connection. The minimum stress values around the hole are quite similar for both cases. However, the maximum stress value decreases from 1304.5 MPa to 746.3 MPa, indicating that the adhesive layer mitigates the stress concentration around the lower-laminate hole, and it also shows that the lower-laminate hole is more sensitive to the mitigating effect of the adhesive layer.

### 4.3. Damage Evolution and Failure Analysis of Connected Structures under Tensile Loading

**Figure 14:**
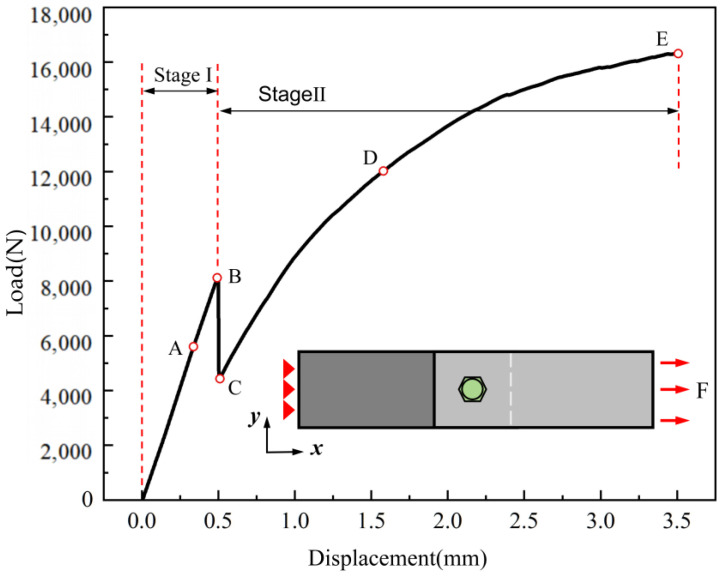
Tensile load–displacement response of composite material adhesive–bolt hybrid interference connection structure.

The tensile load–displacement response curve of the structure with an interference fit of 2.0% is shown in Figure 14 to reveal the overall load-bearing behavior of the typical adhesive–bolt hybrid interference connection structure. The entire load-bearing process can be divided into Stage I and Stage II. For the first half of the curve (Stage I), the load is shared between the adhesive layer and the bolt, exhibiting a linear increasing trend. The tensile load reaches its peak at around 0.5 mm of displacement, followed by a sudden drop, indicating complete failure of the adhesive layer. In the latter half of the curve (Stage II), the load is entirely transferred by the bolt, and the tensile load continues to increase linearly. With the increase in displacement, the bolt’s torsional angle increases, and localized damage begins to occur in the connection hole wall, gradually reducing the stiffness. As the damaged area continues to expand and accumulate, the tensile load eventually reaches the ultimate strength of the structure.

**Figure 15:**
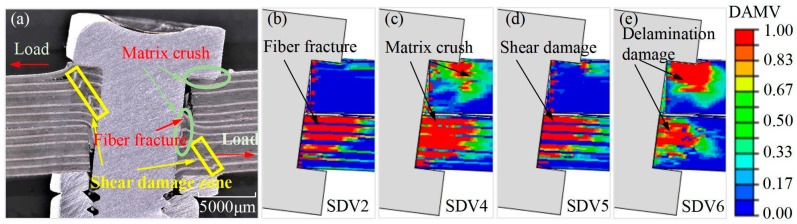
Comparison of deformation and damage simulation of composite material adhesive–bolt hybrid interference connection structure with experimental results: (**a**) experimental results; (**b**) fiber damage; (**c**) matrix damage; (**d**) fiber–matrix shear damage; (**e**) delamination damage.

Figure 15 shows a comparison between the microstructural appearance of the composite adhesive–bolt hybrid interference connection structure simulated using numerical methods and the experimental results regarding deformation and damage. It can be observed that in the direction of tensile loading, the connection hole wall exhibits primary and secondary load-bearing surfaces. The bolt undergoes some torsional rotation due to secondary bending effects, creating a wedge-shaped gap between the composite upper and lower layers and the bolt. As the load increases, the adhesive layer fractures, and it is drawn into the wedge-shaped gap along with the movement of the laminate. The numerical model effectively simulated the deformation and damage state of the structure under tensile loading. Fiber damage (SDV2), matrix damage (SDV4), fiber–matrix shear damage (SDV5), and interlaminar delamination damage (SDV6) are distributed unevenly in the thickness direction. Fiber damage mainly occurs on the primary load-bearing surface, while matrix damage is significantly greater on the primary load-bearing surface than fiber damage. Additionally, matrix damage extends beneath the bolt head. Fiber–matrix shear damage and interlaminar delamination damage are primarily caused by the expansion in the thickness direction due to the bolt’s torsional compression on the connection hole wall. The numerical simulation results demonstrate that the damage prediction model established in this study can effectively reflect the internal damage of the composite adhesive–bolt hybrid interference connection structure under tensile loading.

## 5. Conclusions

This article focuses on the study of composite material bonded–bolted hybrid interference connection structures with the aim of achieving structural strength enhancement. We conducted research on damage to these structures, established a three-dimensional damage prediction model, investigated the load-carrying behavior and failure modes, and revealed the mechanisms by which interference levels affect structural damage and failure. The main conclusions are as follows:

1. The initial damage significantly influences the failure load of the adhesive layer and the composite material. Considering the initial damage around the adhesive layer and composite material during the installation process, the finite element model provides a closer match between the tensile failure load and the experimental results. Therefore, the finite element model should take into account the initial damage to the adhesive layer and composite material.
2. Compared to bolted connections, the adhesive layer mitigates stress concentration around the laminate holes, with the lower laminate being more sensitive to the cushioning effect of the adhesive layer. Under conditions of close contact between the bolt and the hole wall, the tensile strength of the CFRP hybrid bonded–bolted joint, the complete failure load of the adhesive layer, and the quality of the interference at the bolt–hole interface are positively correlated. Specifically, when the interference-fit size is 1.1%, the tensile strength is maximum, followed by interference-fit sizes of 0.5% and 2.0%.
3. In the initial load-bearing stage, the load is primarily transmitted through the adhesive layer, and damage around the composite material hole is not significant. After the complete failure of the adhesive layer, damage around the composite material hole rapidly intensifies, with matrix compression damage being greater than fiber compression damage. Delamination damage is mainly distributed on the upper and lower surfaces of the composite laminate.

## Figures and Tables

**Table 1:** Material properties of CFRP laminates [35].

**E1/GPa**	**E2/GPa**	**E3/GPa**	**G12/MPa**	**G13/MPa**	**G23/MPa**
115	7.8	7.8	4160	4160	2671
**v12**	**v13**	**v23**	**XT/MPa**	**XC/MPa**	**YT/MPa**
0.32	0.32	0.46	2300	1050	50.1
**YC/MPa**	**ZT/MPa**	**ZC/MPa**	**S12/MPa**	**S13/MPa**	**S23/MPa**
183	50.1	183	83.9	83.9	83.9

**Table 2:** Material properties of the type of bolt used.

Material	Elastic Modulus/GPa	Poisson’s Ratio	Tensile Strength/MPa
Ti-6Al-4V	113.8	0.342	896

**Table 3:** Material properties of Ergo7200 [36].

$K_{n}$ **/MPa**	$K_{s}$ **/MPa**	$K_{t}$ **/MPa**	$t_{n}^{0}$ **/MPa**	$t_{s}^{0}$ **/MPa**	$t_{t}^{0}$ **/MPa**
2875	960	960	8	18	18
$G_{I}^{C}$ **/J·mm^−2^**		$G_{\mathrm{II}}^{C}$ **/J·mm^−2^**		$G_{\mathrm{III}}^{C}$ **/J·mm^−2^**	
0.08		0.32		0.32	

**Table 4:** Property degradation rules.

Failure Mode	E1	E2	E3	v12	v13	v23	G12	G13	G23
Fiber tensile failure $\left(\sigma_{11}\geq 0\right)$	0.07	1	1	0.1	0.1	1	0.2	0.2	1
Fiber compression failure $\left(\sigma_{11}<0\right)$	0.14	1	1	0.1	0.1	1	0.2	0.2	1
Matrix tensile failure $\left(\sigma_{22}+\sigma_{33}\geq 0\right)$	0.2	1	1	0.2	1	0.2	0.2	1	0.2
Matrix compression failure $\left(\sigma_{22}+\sigma_{33}<0\right)$	0.4	1	1	0.4	1	0.4	0.4	1	0.4
Fiber/matrix shear failure $\left(\sigma_{11}<0\right)$	1	1	1	1	1	1	0.15	0.15	1
Transverse tensile failure of the matrix $\left(\sigma_{33}\geq 0\right)$	1	1	0.2	1	0.2	0.2	1	0.2	0.2
Transverse compressive failure of the matrix. $\left(\sigma_{33}<0\right)$	1	1	0.2	1	0.2	0.2	1	0.2	0.2

## Data Availability

Data is contained within the article.
